# Physico-chemical and cytotoxic analysis of a novel large molecular weight bacteriocin produced by *Lactobacillus casei* TA0021

**Published:** 2019-10

**Authors:** Elham Noroozi, Naheed Mojgani, Elahe Motevaseli, Mohammad Hossein Modarressi, Majid Tebianian

**Affiliations:** 1 Department of Cellular Molecular Biology, Tehran Science and Research Branch, Islamic Azad University, Tehran, Iran; 2 Department of Biotechnology, Razi Vaccine and Serum Research Institute, Agricultural Research Education and Extension Organization (AREEO), Karaj, Iran; 3 Department of Medical Biotechnology, School of Advanced Medical Technologies, Tehran University of Medical Sciences, Tehran, Iran; 4 Department of Medical Genetics, Tehran University of Medical Sciences, Tehran, Iran

**Keywords:** Bacteriocin, Antimicrobial, *Lactobacillus casei*, Cytotoxicity, Heat labile

## Abstract

**Background and Objectives:**

Antimicrobial peptides produced by lactic acid bacteria have gained enormous attention owing to their health benefits. This study aimed to isolate, purify and characterize the antibacterial protein produced by autochthonous *Lactobacillus casei* TA0021 strain.

**Materials and Methods:**

The antagonistic activity of *L. casei* TA0021 against a number of pathogenic bacteria was tested by agar well diffusion assay. The antimicrobial agent in the neutralized supernatant fluids was subjected to the action of proteolytic enzymes, catalase, lipase and lysozyme, and their tolerance to variable pH and temperature was estimated. The proteinaceous antagonistic compound was precipitated by 60% w/v ammonium sulphate, desalted and subjected to cation exchange and gel filtration chromatography. Approximate molecular weight of Lactocin was determined by SDS-PAGE and non-denaturing gel electrophoresis. Hemoglobin release assay and cytotoxicity effect of Lactocin TA0021 was determined. The results were statistically analyzed.

**Results:**

The antagonistic agent active against *Salmonella* Typhimurium and *Shigella flexneri* appeared resistant to catalase and lipase treatments, while sensitive to the tested proteolytic enzymes. Lactocin TA0021 resisted acidic pH values of 3.0, while alkaline pH values of >9 completely destroyed the activity. The antibacterial peptide was approximately 68 KDa and heat labile as lost its activity at 100°C after 5 minutes. The bacteriocin was non-toxic to MRC-5 cell lines and non-hemolytic. Purification method lead to increase in antibacterial activity while, subsequent decrease in recovery and yield was observed with increasing purification fold.

**Conclusion:**

The purified antimicrobial protein from *L. casei* TA0021 might be used for application in medicinal and food products.

## INTRODUCTION

Many lactic acid bacteria (LAB) produce a high diversity of different antibacterial substances which are defined as bacteriocins or bacteriocin like substances (BLIS) (1–3). These antibacterial substances are a heterogeneous group of anti-bacterial proteins that vary in spectrum of activity, mode of action, molecular weight, genetic origin and biochemical properties. Bacteriocins are cationic peptides that are produced by almost all groups of bacteria. Most bacteriocins are extremely potent, exhibiting anti-microbial activity at nano-molar concentrations, the producer cells are immune to their own bacteriocins (4–6).

Use of chemical preservatives employed in food products to limit the number of pathogenic microorganisms capable of growing within foods has led to increased consumer awareness of the potential health risks associated with some of these substances. In this context, the bacteriocins isolated from lactic acid bacteria (LAB) are reported as GRAS (generally regarded as safe) microorganisms and hence their use in food products pose no health threats besides preventing the growth of pathogenic bacteria (6, 7). Bacteriocin-producing strains may be used as protective cultures to improve the microbial safety of foods. The crude or purified form of these antimicrobial agents may also be applied directly as food preservative. To date nisin, a bacteriocin produced by a food grade bacteria named *Lactococcus lactis*, has found wide application in the food industry where it is used as an effective and safe food bio-preservative (8, 9). Owing to the presence of unusual amino acid lanthionine, nisin is classified as an antimicrobial compound belonging to the group lantibiotics (10).

Different strategies have been applied to purify bacteriocins and have high recovery and yields. Usually multiple purification steps are performed to achieve significant recovery (11). In this study we aimed to purify and characterize the bacteriocin produced by a local isolate *L. casei* TA0021 strain and to assess its future applicability as a biological preservative.

## MATERIALS AND METHODS

### Bacterial growth conditions

*L. casei* TA0021 strain (RTCC 1296-2) was obtained from Razi Type Culture Collection, Razi vaccine and Serum Research Institute (RVSRI), Iran. This strain was isolated from dairy product and was deposited as probiotic bacterial culture at RVSRI. The mentioned bacterial strain was grown in DeMan Rogosa and Sharpe (MRS, HiMedia India) broth at 37°C for 24–48 h in anaerobic jars, while other pathogenic bacteria used in study were grown in Brain Heart Infusion (BHI, HiMedia-India) broth at 37°C for 18–24 h under aerobic condition. All used bacterial cultures were maintained at 4°C and renewed every week for short-term preservation. The long-term conservation of the purified isolates was carried out in MRS broth with sterile glycerol (20%) and stored at −70°C.

### Antimicrobial spectrum

The antimicrobial effects of *L. casei* TA0021 against Gram positive and negative pathogens were examined by agar well diffusion method (6, 12). A number of pathogens namely *Shigella flexneri* (RTCC 1863), *Streptococcus pyogenes* (RTCC 2079), *Staphylococcus aureus* (RTCC 1907), *Salmonella* Typhimurium (RTCC 1679) and *Salmonella* Enteritidis (RTCC 1621) were used as indicator culture in the study. Overnight grown producer cultures were centrifuged at 6000 × g for 30 min and the culture supernatants were filtered using membrane filtration (0.22 μm, Millipore, USA). The indicator culture were adjusted to McFarland Index 0.5 and inoculated into 6 ml of molten soft agar and overlaid on solidified agar plates. Wells were made into the overlaid plates with sterile crock borer and 100 μl of the supernatant fluid collected from the producer culture were added to the individual wells. After 24 h of incubation at 37°C, the plates were observed for the appearance of clear zone around the wells. The zone diameters were measured and recorded in millimeters (mm).

### Bacteriophage assay

In order to negate the presence of phage for the antibacterial activity, the recently described method of Hockett and Baltrus (13) was adopted with slight modifications. Soft agar overlay technique was used by serially diluting the supernatant fluids of the producer culture and spotting 10 μl of the samples onto seeded soft agar overlaid on MRS agar plates. After 24 h of incubation at 37°C the plates were examined for the presence of plaques.

### Bacteriocin identification

One liter of supplemented MRS (3% maltodextrose and 0.2% Tween 80) broth was inoculated with the producer cultures and incubated overnight at 37°C for 24 h. The cell free extracts were collected by centrifuging the culture fluids at 6000×g, for 20 min at 4°C. The supernatant fluid (SF) were neutralized to pH 6.5 and filtered using 0.22 μm filter membranes (Millipore, USA) and their antibacterial activity evaluated by agar well diffusion assay.

In order to identify the protein antagonistic agent as bacteriocin, the collected neutralized supernatant fluid (NSF) were treated with catalase, lysozyme, lipase, pronase E, trypsin and proteinase K (Sigma, USA) at a final concentrations of 1 mg/ml in phosphate buffer (pH 7.0), respectively. The enzyme treated fractions were analyzed for the remaining antibacterial activity after 4 h of incubation at 37°C by agar well diffusion assay.

### Bacteriocin characterization

The NSF of the producer were pH adjusted to 2.0 to 11.0 with 4 N hydrocholoric acid (HCl) and or 3N sodium Hydroxide (NaOH) and the remaining activity in the fractions was determined at different time intervals by agar well diffusion assay. *S. flexneri* was used as indicator strain in all following experiments owing to its maximum sensitivity to the tested bacteriocin.

Thermostability of the antagonistic compound produced by the selected isolates was evaluated by heating the supernatant fluid at 40, 60, 80, and 100°C and determining the residual activity after every 10 min of time intervals for maximum 90 min by the method described earlier.

### Bacteriocin quantification

The critical dilution assay described previously (6, 14) was used to quantify the inhibitory activity exhibited by the NSF of the mentioned producer strains against the indicator strains. A serial two fold dilution of neutralized supernatant fluid in MRS broth was assayed for residual activity as described earlier. The activity was recorded in arbitrary units per ml (AU/ml), defined as the reciprocal of the highest dilution demonstrating discernible activity.

### Bacteriocin purification and concentration

The antagonistic substance produced by the selected isolates was partially purified by subjecting the supernatant fluid (pH 6.5) of the producer strains to ammonium sulfate precipitation at final concentrations of 60% w/v (15, 16). The precipitate were recovered by centrifugation (10,000×g, 25 min, 4°C), re-suspended in sterile MilliQ water and dialyzed against sterile MilliQ water at 8°C for 24 h, using 5KDa dialysis tubings (Millipore, USA) for desalting the obtained protein precipitates. Traces of ammonium sulphate were further removed by passing the fraction through Sephadex G-25 columns equilibrated with 20 mM sodium phosphate (pH 6.8)

Most bacteriocins produced by Lactic Acid Bacteria (LAB) are known to bear positive charges at pH near neutrality, and hence cation exchange resins is considered most appropriate for their purifications. In this study, the NSF (pH 6.0) suspension were subjected to cation-exchange column (Carboxymethyl cellulose glass column 26 × 200nm), washed with a phosphate buffer (pH 6.0) containing sodium chloride (NaCl) gradient (0.1 to 1.0 M). Fractions of 4 ml were collected at a flow rate of 6 mL/hour, and the protein contents were monitored by reading absorbance at 280 nm. The collected fractions were tested for their antibacterial activity using agar-well diffusion method. The fractions showing antibacterial activity were pooled together, filter sterilized (0.22 μm) and subjected to size exclusion (gel filtration) chromatography using Sephadex G-100 (coarse) column (2× 150 cm; Bio-Rad) equilibrated with 50 mM phosphate buffer (pH 6.0) and eluted with the same buffer. All collected fractions with the antibacterial activity were pooled together, concentrated using Polyethylene glycol (PEG 6000, Sigma, USA).

### Ultrafiltration

Partially purified lactocin TA0021 was ultrafiltred using 30 KDa (Amicon, USA) ultra-filter membrane tubes using centrifugations (6000×g, 30 min, and 4°C). The collected filtrate and the retentate were analyzed for the antagonistic activity (AU/ml) and protein concentrations (mg/ml) as described earlier. The retentate showing antibacterial activity was sterilized filtered using 0.22 μm filter membranes and stored at 4°C for further use.

### Protein and molecular size estimation

The protein concentrations were determined by the Lowry method using BSA as standard (17). Approximate molecular weights of the antagonistic peptides were determined by SDS-PAGE analysis as described previously (18), using 12% and 15% polyacrylamide concentrations in the stacking and the separating gel, respectively. To determine the location of the bacteriocin band native polyacrylamide gel electrophoresis (12%) was used. Following electrophoresis (60 V for 1 h), the unstained gel was washed extensively with ultra-pure water (milliQ), and then overlaid with the 10^6^ CFU/ml of the indicator strain. The zone of clearance around the respective protein band was observed after 24 h of incubation.

### Cytotoxicity assay

The purified bacteriocin fractions were assessed for their *in vitro* cytotoxicity level on human lung fibroblast (MRC5) cell lines purchased from Pasture Institute, National Cell Bank, Iran. The mentioned cell lines were pre-cultured in tissue culture flasks containing Dulbecco modified eagle medium (DMEM) medium (Sigma, Poole, United Kingdom) with 10% (v/v) fetal bovine serum (FBS), and 1% penicillin/streptomycin (Invitrogen, USA). The culture flasks were maintained in a humidified 37°C atmosphere with 5% CO_2_. Once adapted, the cells were routinely passaged in the appropriate culture medium until the cell monolayer reached 80% confluence. Cell counts were made in a Neubauer haemocytometer, while the cell viability was assayed using trypan blue dye exclusion method (19).

For cytotoxicity assay, working solutions of Lactocin TA0021 and Colicin E1 (sigma, USA) was prepared in the respective medium and a total of 10^4^ of the mentioned cell lines seeded in 96 well plates, respectively. The wells were treated with different concentrations of the bacteriocin (0.25–4 μg/ml) preparations. The plates were incubated for 72 hours at 37°C under 5% (v/v) CO_2_ and MTT [3-(4, 5-dimethylthiazole-2-yl)-2, 5-diphenyltetrazolium bromide] colorimetric assay was used to measure the inhibitory effect of the purified bacteriocin and Colicin E1 suspensions on the growth of respective cell line (20). The cell viability percentage of the cell lines after treatment with Lactocin TA0021 was determined according to the mentioned (21):

Viability (percentage of control) = [(absorbance sample-absorbance blank / [absorbance control-absorbance blank)] * 100

Moreover, the IC50 (concentrations of the bacteriocin that inhibits 50% of the cell viability after 72 h of exposure) was estimated according to the protocol described earlier (22).

### Hemolytic activity

Hemolytic activity of Lactocin TA0021 and Colicin E1 (Sigma, USA) was determined by hemoglobin release assay which is monitored by reading the OD at 414nm of microtiter plates inoculated with sheep erythrocytes and the mentioned bacteriocins (23). Hemolysis percentage was calculated by the given formula:

Hemolysis (%) = (A_414_ of treated supernatant * A_414_ in PBS) / (A_414_ in 0.1 % Triton X-100 * A_414_ in PBS) * 100

PBS was used as negative control (0% hemolysis), while 0.1 % Triton X-100 was used as positive control (100 % hemolysis). The results were expressed in means ± SD of three independent experiments.

### Statistical analysis

All tests were performed in triplicate to ensure reproducibility and the results were interpreted as means ± SD of three independent experiments. Variance analysis was determined with mean percentages of viability, and significance differences were determined by ×2 test with 5% probability (P< 0.05).

## RESULTS

**Fig. 1:**
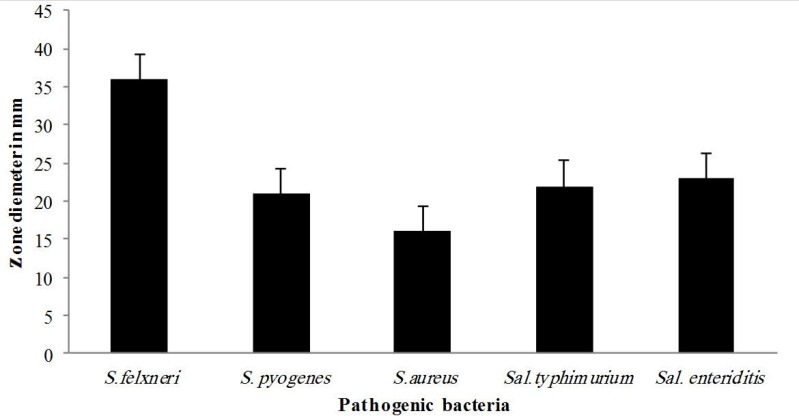
Antibacterial activity of neutralized supernatant fluid of *L. casei* TA0021 against the tested pathogenic bacteria by agar well diffusion assay

In this study, the bacteriocin produced by *L. casei* TA0021 was isolated and identified following purification and physic-chemical characterization. As observed in Fig. 1, the studied *Lactobacillus* strain demonstrated antagonistic activity against all tested bacterial pathogens. *S. flexneri* appeared to be the most sensitive strain followed by *S.* Enteriditis, *S.* Typhimurium, *S. pyogens* and *S. aureus*.

**Fig. 2:**
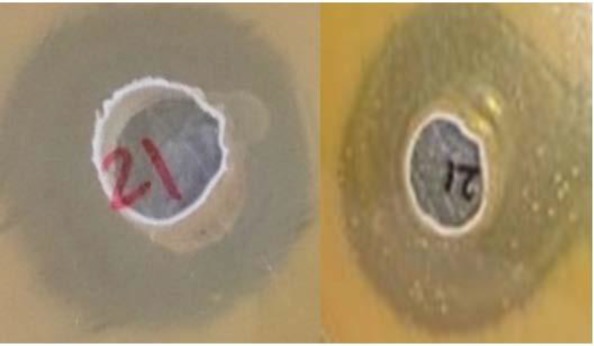
Antibacterial activity of neutralized supernatant fluid 50 of *L. casei* TA0021 against *S. flexneri* observed by agar well 60 diffusion assay

As seen in Fig. 2, *S. flexneri* showed maximum inhibitory zone (36 mm), while least resistant strain was *S. aureus* as an inhibitory zone of only 17 mm was observed around the wells during agar well diffusion assay.

During physic-chemical investigations, the antagonistic compound was resistance to pH neutralization, while showed sensitivity to the tested proteolytic enzymes as their inhibitory effect was completely demolished after subjection to trypsin, pronase E and proteinase K enzymes. Hydrogen peroxide and bacteriophage were not responsible for the observed antibacterial activity as catalase treated neutralized supernatant fluid retained their activity and no plaques were observed during bacteriophage assay, respectively. Additionally, the antagonistic compound was unaffected by the enzymes lipase and lysozyme which indicates the absence of glycol-lipid moiety in the protein bacteriocin molecule. The protein antagonistic compound was named Lactocin TA0021 hence forward.

Thermo-stability of Lactocin TA0021 was tested which indicated variable resistance of the target protein to temperatures ranging from 40°C to 100°C. As depicted in Table 1, the protein antagonistic compound was fully resistant to temperatures of 40°C and 60°C for 90 min. However, with increasing exposure time the inhibitory activity showed gradual decrease. The activity was completely lost after 10 min at boiling temperatures.

**Table 1:** Thermo-stability of crude Lactocin TA0021 at different time intervals

**Time (min)**	**Temperature**			

	**40°C**	**60°C**	**80°C**	**100°C**
0	++++	++++	++++	++++
10	++++	++++	+++	+
20	++++	++++	++	−
30	++++	++++	++	−
40	++++	+++	+	−
50	++++	++	+	−
60	++++	++	−	−
70	+++	++	−	−
80	+++	++	−	−
90	++	++	−	−

Zone diameters expressed as ++++=>25 mm; +++= 18–24 mm; ++= 12–17 mm; += <12 mm; −+ no zone of inhibition

The protein appeared stable at acidic pH values of 3 in contrast to its sensitivity to alkaline pH values of 9. Maximum inhibitory activity (2560 AU/ml) was recorded at pH ranging from 5.0 to 7.0, while no activity was recorded at extreme pH values of above 9.0.

In order to purify the target protein (Lactocin TA0021), the neutralized supernatant fluids were subjected to 60% ammonium sulphate at 4°C. Maximum amount of the protein precipitated in the fractions were further desalted by dialysis and later fractionated by gel filtration and cation-exchange chromatography. The overall yield and activity are summarized in Table 2. The cell free supernatant was concentrated approximately 25 fold by 60% ammonium sulphate, which consequently resulted in significant increase in specific activity with recovery yield of approximately 90%. During subsequent purification stages, the specific activity of Lactocin TA0021 increased by 200 to 250-fold and the recovery was 60–65% (following cation exchange chromatography and gel filtrations. By Gel filtration, the activity was eluted as a distinct peak corresponding to a molecular weight above 60000. The specific activity increased 250 to 342 fold with final recovery of 37%.

**Table 2:** Summary of the purification steps of Lactocin Ta0021 from the culture supernatant fluid of *L. casei* TA0021

**Purification Stage**	**Culture supernatant**	**60% Ammonium sulphate saturation**	**Cation-exchange chromatography**	**Gel filtration (SephafdexG-100)**	**PEG 6000 Conc**
Volume (ml)	100	55	16	12	3.5
Activity (AU /ml)	2560	2295	1688	1540	960
Total protein (mg)	18.36	12.32	7.41	6.18	2.88
Specific activity	139.4	186.2	227.8	249.1	342.8
Yield (%)	100	89.96	65.93	60.15	37.50

Specific Activity (AU/mg) = Total activity of the subsequent purification step/Total protein of the same step; Yield (%) = Total activity of the subsequent purification step/Total activity in the crude culture supernatant * 100.

**Fig. 3:**
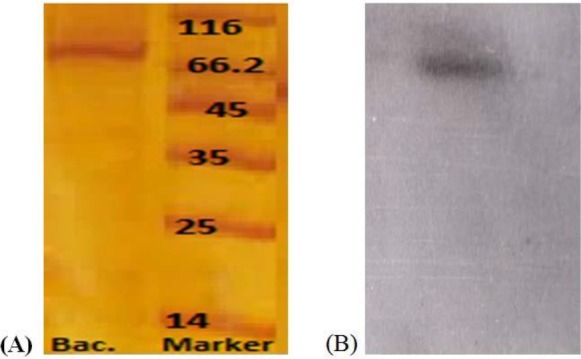
Approximate molecular weight of Lactocin TA0021 on 12% SDS-PAGE (A) and on non-denaturing gel (B).

The partially purified bacteriocin fractions were subjected to ultrafiltration using 30KDa ultra-filter tubes. The activity was completely recovered in the retentate after centrifugation, and no activity was seen in the filtrate. These results indicated the presence of a protein molecule greater than 30KDa. Following these studies, the approximate molecular weight of the bacteriocin was estimated by 12% SDS-PAGE analysis. A protein bond of approximately 68 KDa was shown to be responsible for the Lactocin TA0021 bacteriocin (Fig. 3). While, non-denaturing gel confirmed the respective protein band related to Lactocin TA0021.

The cytotoxic effect of Lactocin TA0021 and Colicin E1 was determined against MRC5, a human lung fibroblast cell line. As depicted in Table 3, the cell viability of the cell line decreased with increasing concentrations of Lactocin TA0021 and Colicin E1. A dose response effect was observed as with increasing concentrations of the respective bacteriocins a decrease in cell viability was recorded. Highest used concentrations of the bacteriocins (4 μg/ml) resulted in more than 50% destruction of the cell viability. While, approximately 2 μg/ml of the bacteriocins resulted in only 20% loss in the cell viability of MRC5 cell lines with Colicin E1 exhibiting lower cytotoxic effects than Lactocin TA0021 on the respective human cell lines.

**Table 3:** Cytotoxic effect of variable concentrations of Lactocin TA0021 and Colicin E1 on MRC5 cell lines estimated in terms of cell viability percentage

**Bacteriocin**	**Concentrations (μg/ml)**								

	**0.25**	**0.5**	**1.0**	**1.5**	**2.0**	**2.5**	**3.0**	**3.5**	**4.0**
Colicin	9 9.1 ± 1.11	94.5 ± 0.37	90.3 ± 0.51	85.5 ± 0.73	81.0 ± 0.9	71.1 ± 0.2	52.9 ± 0.22	48.4 ± 0.18	41.6 ± 0.43
TA0021	96.1 ± 0.66	93.1 ± 0.68	87.7 ± 0.8	82 ± 0.45	77.9 ± 0.29	70 ± 0.32	58.5 ± 0.47	51.3 ± 0.6	44.1 ± 0.8

Colicin: Colicin E1; TA0021: Lactocin TA0021 isolated and purified from *L. casei* TA0021 in this study. All values are expressed as mean ± SD of three results.

The hemolytic activity of the Lactocin TA0021 and Colicin E1 was estimated based on the percentage lysis of sheep erythrocytes within 2 hours of exposure time. According to the obtained data, both the bacteriocins had almost similar hemolytic pattern with relative hemolysis lower than 3.5%. Compared to trition X-100 (0.1% v/v) which caused 100% hemolysis, the hemolysis caused by Lactocin TA0021 and Colicin at the highest used on concentrations was approximately 3.29% to 3.43%, respectively.

## DISCUSSION

Bacteriocins produced by different species of lactic acid bacteria (LAB) are among the most studied proteins. They are considered potent bio-preservative agents and their application in food industry is currently the subject of extensive research (16). So far, nisin and pediocin PA-1 are bacteriocins licensed for use in certain foods as preservatives (16). To date, a number of bacteriocinogenic lactobacilli have been purified and characterized in order to better understand these molecules and to improve their efficacy to serve mankind. In this study, Lactocin TA0021, an antimicrobial molecule of high molecular weight (68KDa) secreted by a *L. casei* strain TA0021 was purified and biologically characterized.

One of the salient features of bacteriocinogenic LAB for application in food and as a probiotic candidate is their ability to inhibit the growth of unwanted spoilage or pathogenic microorganisms. The intragenic bacteriocinogenic potential of *L. casei* TA00021 was checked by exposing the strain to other pathogenic bacteria and observing zone of inhibition in agar well diffusion assay. Similar to many reported bacteriocins (24) the Lactocin in study inhibited the growth of a number of Gram positive and Gram negative pathogens.

Antagonism seen in *Lactobacillus* species due to metabolic end products such as acid and hydrogen peroxide might be erroneously attributed to the production of bacteriocin like compounds. Beside these metabolites, sometimes bacteriophages could also present antagonistic effects similar to that of bacteriocins (25). Hence, it is essential to carefully characterize antagonistic compound following a number of physic-chemical studies. In this respect, complete inactivation of the activity was observed when the supernatant fluid of *L. casei* TA0021 was treated with proteolytic enzymes like proteinase K, trypsin and pronase, thus confirming its proteinaceous nature. During the course of this study, the antibacterial moiety of Lactocin TA0021 was recorded as simple protein molecule without carbohydrate or lipid moiety involvement in the bioactivity of the bacteriocin. Similarly, Sakacin A, Plantacin B, Lacticin A and B have been shown as un-conjugated protein molecules with no involvement with carbohydrates or lipids (26–30).

Owing to the importance of bacteriocins in food and other products of human interests, a number of salient features for their application are essentially required to be investigated. In this context, pH and temperature stability are the most studied parameters (31). During these analysis, the bacteriocin in this study appeared to be stable within pH range of 3.0 to 8.0, while extreme alkaline pH values exceeding pH9.0 were detrimental to the proteinaceous compound.

An important criteria for classifying bacteriocins is based on their thermal resistance (32, 33). Based on the proposed classification scheme, Lactocin TA0021 appeared a heat labile bacteriocin belonging to class III. Other representative of this class are Helveticin J, Acidophilucin A, Lactacin A,B and Caseicin 80 (27–29, 33, 34).

Most of the LAB bacteriocins are defined as short cationic polypeptides which seldom contain more than 60 amino acids, with some hydrophobic characteristics (35). In this study, we isolated and purified Lactocin TA0021 different purification strategies starting from ammonium sulphate precipitation, gel filtration to cation exchange chromatography. Protein precipitations by ammonium sulphate is considered one of the simplest and cost effective method that can fractionate proteins of different molecular weight (35, 36). During the precipitation of Lactocin TA0021 by the mentioned salt, some amount of the protein was precipitated as a surface pellicle that might indicate the association of the bacteriocin molecule with the hydrophobic globular micelle like structure in the supernatant fluid. Similar phenomenon have been reported for other protein bacteriocins like Lactocin S, Lactocin F etc (32, 33). The approximate molecular size of the purified Lactocin TA0021 appeared to be above 30 KDa as seen by ultrafiltration studies implying 30 KDA ultrafilter membranes. These results were confirmed by SDS-PAGE and non-denaturing gels that indicated a protein band of 68 KDa to be responsible for the antibacterial activity. Further efforts to analyze the amino acid sequence of Lactocin TA0021 are underway.

## CONCLUSION

A large heat labile bactriocin produced by locally isolated *L. casei* strain TA0021 was classified as class III bacteriocins. The mentioned bacteriocin named as lactocin TA0021 appeared a safe antibacterial protein as it inhibited the growth of tested pathogens and was neither cytotoxic to normal MRC5 cell lines nor hemolytic to sheep erythrocytes. Based on *in vitro* studies, Lactocin TA0021 might be useful for application in variety of health products aimed to benefit man and animals in future.
